# Anti-Atherosclerotic Effect of Afrocyclamin A against Vascular Smooth Muscle Cells Is Mediated via p38 MAPK Signaling Pathway

**DOI:** 10.22074/cellj.2021.8128

**Published:** 2021-07-17

**Authors:** Yan Gu, Zhanzhan Xiao, Jianlie Wu, Mingjin Guo, Ping Lv, Ning Dou

**Affiliations:** 1 Department of Vascular Surgery, Tianjin First Center Hospital, Tianjin, China; 2 Department of Emergency Services, The Fourth People’s Hospital of Jinan City, Jinan, Shandong Province, China; 3 Department of Vascular Surgery, The Affiliated Hospital of Qingdao University, Qingdao City, China; 4 Department of Hematology, The Fourth People’s Hospital of Jinan City, Jinan, Shandong Province, China; 5 Department of General Surgery, Shanghai Fourth People's Hospital Affiliated to Tongji University School of Medicine, Shanghai, China

This article published in Cell J (Yakhteh), Vol 23, No 2, 2021, on pages 191-198, corresponding author and corresponding address were changed based on authors’ request. The authors would like to apologies for any inconvenience caused.

